# Genome mining of labdane-related diterpenoids: Discovery of the two-enzyme pathway leading to (−)-sandaracopimaradiene in the fungus *Arthrinium sacchari*

**DOI:** 10.3762/bjoc.20.65

**Published:** 2024-04-03

**Authors:** Fumito Sato, Terutaka Sonohara, Shunta Fujiki, Akihiro Sugawara, Yohei Morishita, Taro Ozaki, Teigo Asai

**Affiliations:** 1 Graduate School of Pharmaceutical Sciences, Tohoku University, Sendai 980-8578, Japan

**Keywords:** diterpenoids, fungi, genome mining, labdane, terpene cyclase

## Abstract

Labdane-related diterpenoids (LRDs) in fungi are a pharmaceutically important, but underexplored family of natural products. In the biosynthesis of fungal LRDs, bifunctional terpene cyclases (TCs) consisting of αβγ domains are generally used to synthesize the polycyclic skeletones of LRDs. Herein, we conducted genome mining of LRDs in our fungal genome database and identified a unique pair of TCs, AsPS and AsCPS, in the fungus *Arthrinium sacchari*. AsPS consists of catalytically active α and inactive β domains, whereas AsCPS contains βγ domains and a truncated α domain. Heterologous expression in *Aspergillus oryzae* and biochemical characterization of recombinant proteins demonstrated that AsCPS synthesized copalyl diphosphate and that AsPS then converted it to (−)-sandaracopimaradiene. Since AsPS and AsCPS have distinct domain organizations from those of known fungal TCs and are likely generated through fusion or loss of catalytic domains, our findings provide insight into the evolution of TCs in fungi.

## Introduction

Terpenoids are a structurally diverse family of natural products, including more than 80,000 compounds [1]. In the biosynthesis of terpenoids, terpene cyclases (TCs) add structural diversity and complexity. TCs are generally classified into two main classes, class I and class II. Class I TCs initiate the cyclization by heterolytic cleavage of substrates to generate a diphosphate anion and an allylic carbocation, and class II enzymes start cyclization by protonating a double bond or an epoxide existing in their substrates. From a structural point of view, TCs consist of α, β, and γ domains in various combinations. As these TCs are assumed to be evolved by fusion and loss of domains [2–4], the functional analysis of terpene synthases of unique domain organization is of great significance to understand the evolutionary traits of TCs.

**Figure 1:**
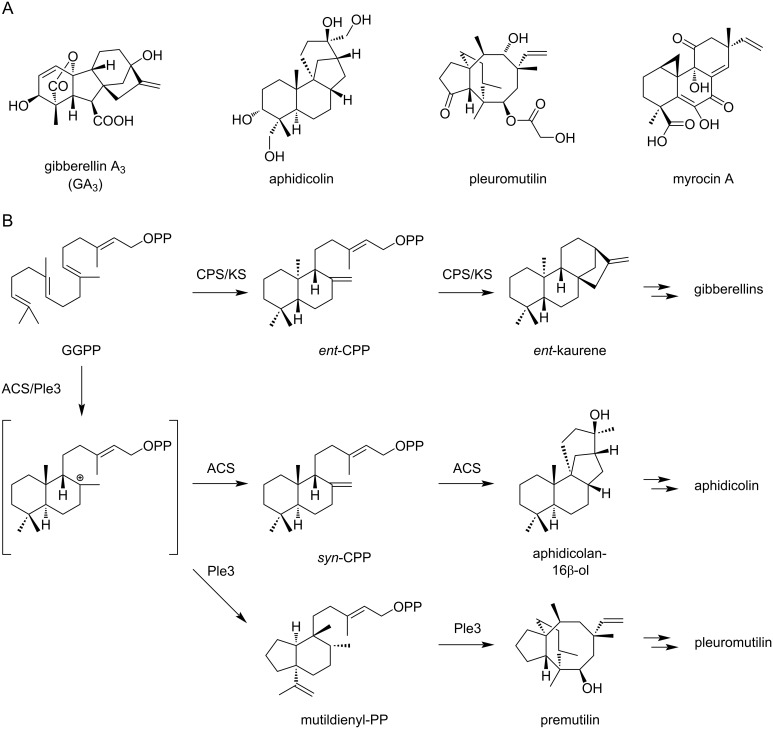
LRDs in fungi. A) Chemical structures of representative fungal LRDs. B) Reactions catalyzed by selected fungal bifunctional TCs.

Among terpenoids, labdane-related diterpenoids (LRDs) are an important class which includes biologically active molecules such as plant hormone gibberellins (Figure 1A). In their biosynthesis, class II TCs often synthesize copalyl diphosphate (CPP) or its stereoisomers, *ent*-CPP and *syn*-CPP. Class II TCs can also generate further structural diversity through hydride shifts, methyl shifts, and/or skeletal rearrangement of the labdadienyl^+^ diphosphate intermediate which is formed by the initial bicyclization. Following this class II TC-mediated cyclization, class I TCs subsequently catalyze the second cyclization to construct the polycyclic scaffold of natural products [5]. In plants, two independent αβγ tri-domain TCs, *ent*-CPP synthase (CPS) and *ent*-kaurene synthase (KS), are often used for this conversion [6], and a single bifunctional enzyme that successively catalyzes these reactions is also known [7]. Bacteria also use two enzyme systems for the biosynthesis of LRDs, but the domain organization of the corresponding TCs is different from those of plant enzymes. In bacteria, the class II enzymes with βγ domains and the class I enzyme with a single α domain are employed [8]. In fungi, only bifunctional enzymes consisting of αβγ tri-domains have been identified to date [9–13]. In the evolutionary aspects, fungal bifunctional TCs are proposed to have been acquired from plants by a horizontal gene transfer event [14] and eukaryotic tri-domain TCs are proposed to be derived from the early fusion of bacterial class I and II enzymes [3,15].

Fungal genomes contain a number of biosynthetic genes that are attractive targets for genome mining of novel natural products and biosynthetic enzymes [16–18]. In our research group, genome-guided exploration of fungal natural products and biosynthetic machineries have been conducted, leading to the discovery of various new natural products and enzymes [19–27]. Although fungal LRDs are a biologically and pharmaceutically important class of natural products including DNA polymerase α inhibitor aphidicolin [28] and a ribosome-targeting antibiotic pleuromutilin [29] (Figure 1), they remained as underexplored targets in genome-mining approaches. In this study, we examined the biosynthetic genes for LRDs and identified two TCs consisting of αβ and αβγ domains. Heterologous expression in *Aspergillus oryzae* and biochemical characterization of recombinant enzymes unveiled a fungal two-enzyme pathway to isopimaradiene, which is unprecedented for fungal LRDs.

## Results and Discussion

**Figure 2:**
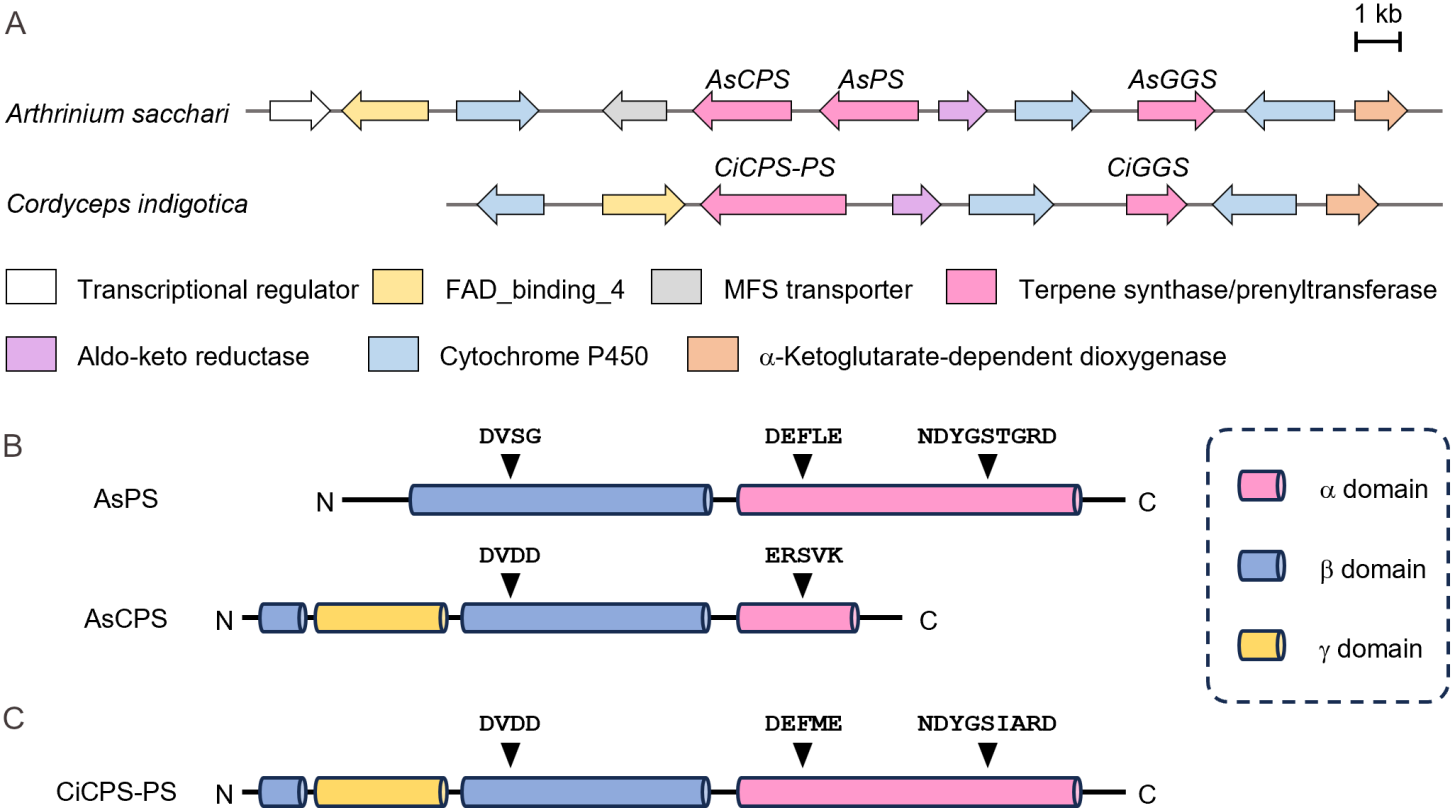
Sequence analysis of AsPS and AsCPS. A) Biosynthetic gene clusters containing AsCPS and AsPS. B) Domain organization of AsPS and AsCPS. C) Domain organization of CiCPS-PS.

To explore the fungal LRDs, we searched our in-house database by BLAST using known fungal bifunctional TCs such as aphidicolan-16β-ol synthase (ACS) as queries. We identified a pair of genes encoding TCs, which we later defined as AsPS (isopimaradiene synthase) and AsCPS, in the genome of *Arthrinium sacchari* Kumo-3 and *Ar. sacchari* MPU169. AsPS and AsCPS are clustered with the putative geranylgeranyl diphosphate synthase gene (AsGGS), suggesting that they are related to the biosynthesis of diterpenes (Figure 2A). AsPS and AsCPS consist of αβ and αβγ domains, respectively. Further sequence analysis revealed that the β domain of AsPS contains a mutation at the catalytic aspartic acid residue, indicating that this enzyme functions as class I TC (Figure 2B and Figure S1 in Supporting Information File 1). AsCPS was assumed to be a class II TC because its α domain is truncated and is likely nonfunctional. Given these domain organizations of identified TCs, we hypothesized that AsPS and AsCPS would likely cooperate to synthesize a diterpene skeleton: After the initial cyclization of GGPP possibly catalyzed by a putative class II enzyme AsCPS, the resulting labdane or related skeleton would undergo cyclization by a putative class I enzyme AsPS. To assess this hypothesis, we conducted the functional characterization of AsPS and AsCPS as described below.

**Figure 3:**
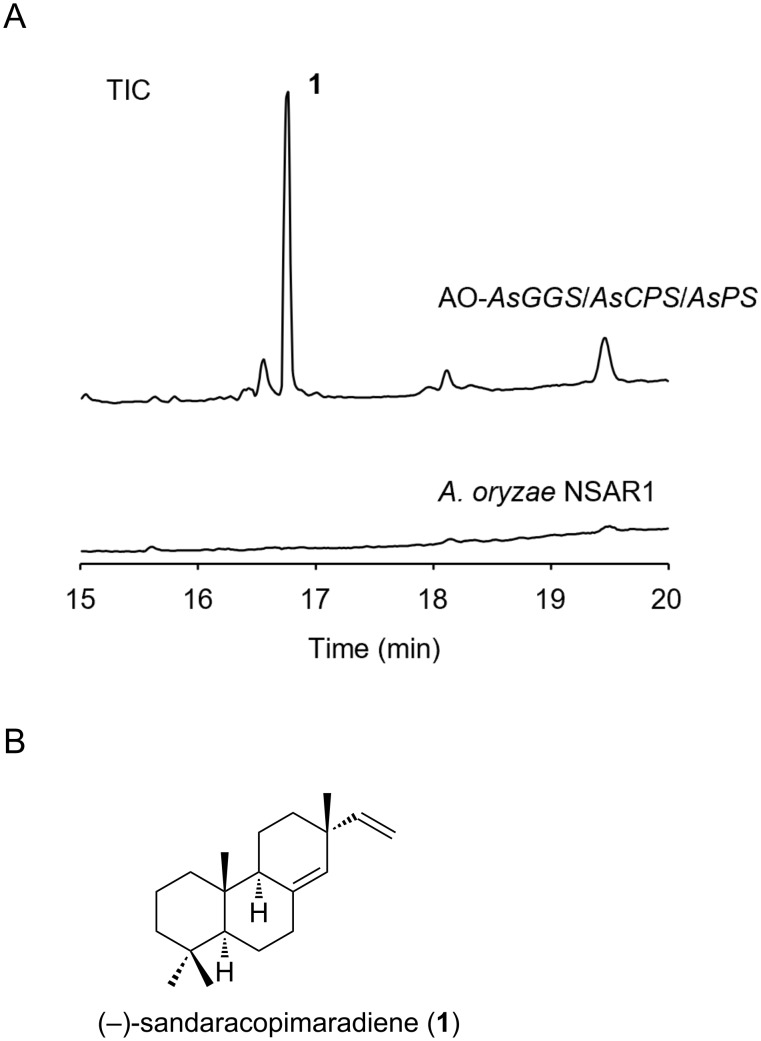
GC–MS analysis of the *A. oryzae* transformant. A) TIC of the extracts obtained from AO-*AsGGS*/*AsCPS*/*AsPS* and *A. oryzae* NSAR1. Compound **1** was identified to be a product, while the other minor peaks were not related to diterpenoids and were likely derived from the host strain. B) Chemical structure of (−)-sandaracopimaradiene (**1**)).

First, we examined the heterologous expression of *AsPS* and *AsCPS* in the heterologous host *Aspergillus oryzae* NSAR1. These two genes along with *AsGGS* were amplified and integrated into the pUARA2 vector, yielding the plasmid pUARA2-*AsGGS*/*AsCPS*/*AsPS* (Table S1 in Supporting Information File 1). The constructed plasmid was introduced into *A. oryzae* to give the transformant AO-*AsGGS*/*AsCPS*/*AsPS*. We then analyzed the metabolite of this transformant by GC–MS. By comparing the metabolites extracted from *A. oryzae* NSAR1, we identified compound **1** as a product (Figure 3A and Figure S2 in Supporting Information File 1). We also examined the expression of several oxidoreductases in the flanking region in the same host strain, but the possible products other than **1** were not clearly observed (data not shown). Further characterization of oxidative modifications is in progress and will be reported elsewhere.

We successfully purified compound **1** from the mycelial extract of the *A. oryzae* transformant harboring *AsGGS*, *AsPS*, and *AsCPS* with a yield of 10.1 mg. The chemical formula of **1** was elucidated to be C_20_H_32_ due to the monoisotopic ion peak at *m*/*z* 272.2494 [M]^+·^ (calcd for C_20_H_32_, 272.2499) in the HREIMS spectrum. The ^1^H and ^13^C NMR spectra of **1** were identical to those of sandaracopimaradiene [30–31]. As the specific rotation of compound **1** was in good agreement with the reported data ([α]_D_^24^ −14.3 (*c* 0.97, CHCl_3_) in this study; [α]_D_^25^ −10.29 (*c* 0.65, CHCl_3_) in the literature [31]), compound **1** was determined to be (−)-sandaracopimaradiene (Figure 3B).

**Figure 4:**
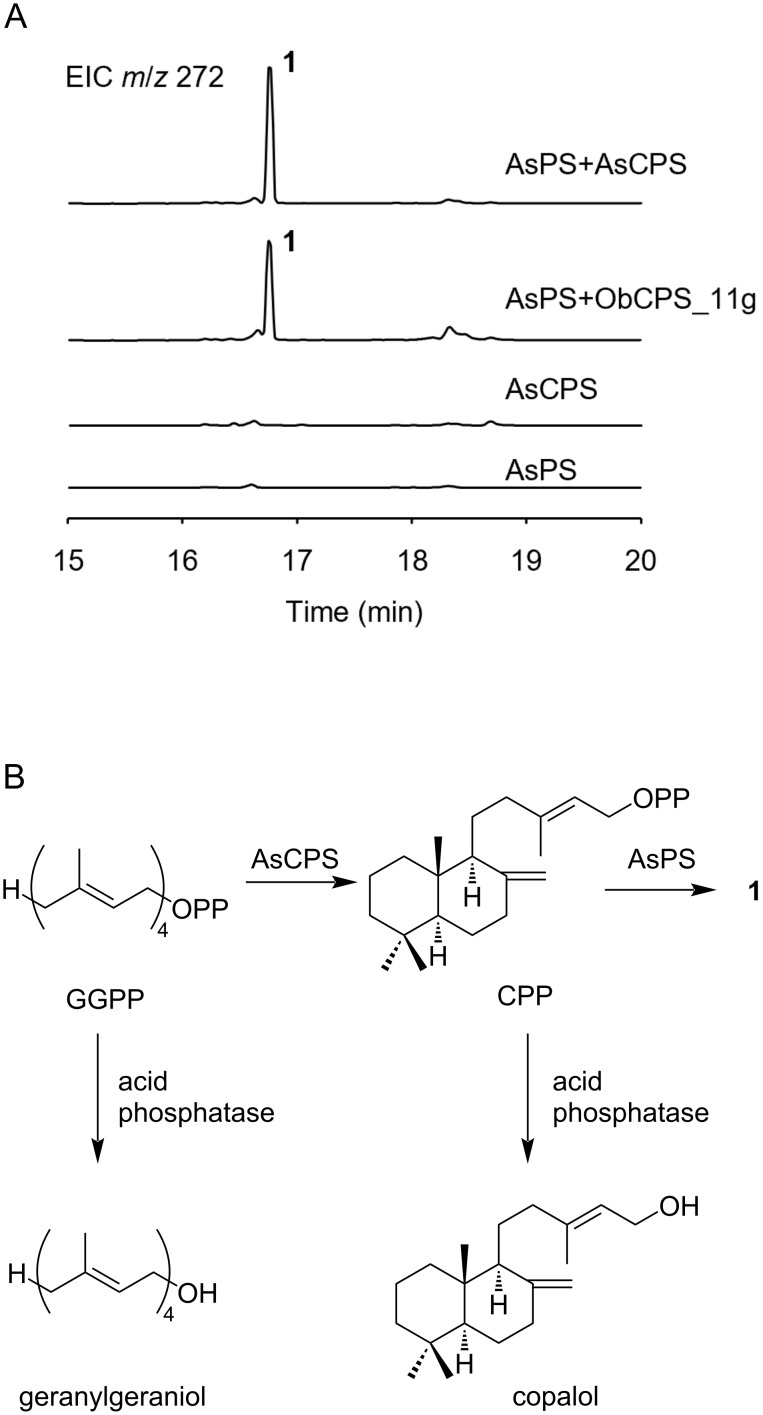
GC–MS analysis of the enzymatic reactions. A) Extracted ion chromatograms (EICs) at *m*/*z* 272 of the extracts from the reaction mixture of AsPS and AsCPS; AsPS and ObCPS_11g are shown. B) Proposed reactions catalyzed by AsPS, AsCPS, and acid phosphatase.

We then turned our attention to the individual function of these enzymes. With this aim, AsPS and AsCPS were expressed in *Escherichia coli* as N-terminal hexa-histidine-tagged proteins and purified by Ni-affinity chromatography (see Supporting Information File 1, Figure S3). The purified proteins were incubated in the presence of GGPP and Mg^2+^. GC–MS analysis of the hexane extract revealed the enzyme-dependent formation of compound **1** (Figure 4A and Figure S2 in Supporting Information File 1). The formation of **1** was not observed when either AsPS or AsCPS was omitted from the reaction mixture, showing that both enzymes were indispensable for the biosynthesis of compound **1**.

Based on the abovementioned results and domain organizations of AsPS and AsCPS, we hypothesized that compound **1** was synthesized via *normal*-CPP. We incubated AsPS with the cell-free extract of *E. coli* expressing ObCPS_11g, which synthesizes *normal*-CPP [32]. As expected, the formation of **1** was observed (Figure 4A and Figure S2 in Supporting Information File 1), indicating that the product of AsCPS was the same as that of ObCPS_11g. In addition,we incubated AsCPS in the presence of GGPP, followed by hydrolysis by acid phosphatase. GC–MS analysis revealed that AsCPS synthesizes the same product to ObCPS_11g (see Supporting Information File 1, Figure S4A and B). It should be noted that the stereochemistry of CPP needs further verification due to the lack of chiral resolution of our GC–MS conditions. Based on these results, we proposed that AsCPS synthesizes CPP and that AsPS subsequently converted it to compound **1**.

**Figure 5:**
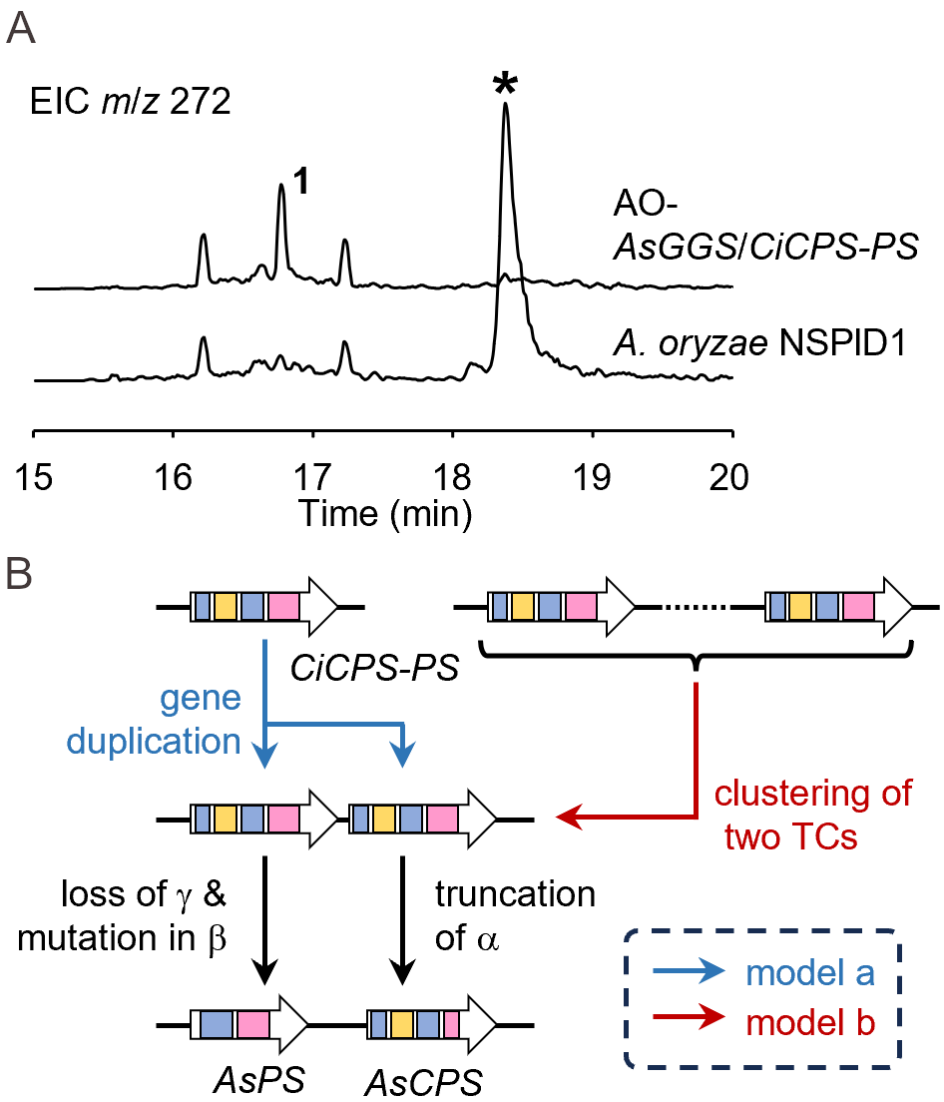
Characterization of CiCPS-PS. A) GC–MS analysis of the transformant expressing *CiCPS-PS* and *AsGGS*. EICs at *m*/*z* 272 are shown. The peak marked by the asterisk is probably derived from the host strain. B) Putative models of evolutionary relationships of CiCPS-PS, AsPS, and AsCPS.

Notably, *Ar. sacchari* MPU169 is known as a producer of myrocins (Figure 1A), which are known to exhibit antiangiogenic activity [33]. Based on the chemical structure and absolute configuration of **1**, we assumed this compound could be a biosynthetic precursor of myrocins. In the flanking regions of *AsPS* and *AsCPS*, genes encoding oxidoreductases such as cytochrome P450 are present. These genes might be involved in the oxidation of **1** in the biosynthesis of myrocins. A similar gene cluster was also found in the genome of *Cordyceps indigotica* (Figure 2A). In this cluster, one bifunctional TC, which we later defined as CiCPS-PS, was present instead of separate TCs, such as AsCPS and AsPS. Although we could identify similar BGCs containing one bifunctional TCs in the public database, there are no similar BGCs with separate type TCs (Figure S5 in Supporting Information File 1). Heterologous expression of CiCPS-PS with AsGGS also gave **1** as a product (Figure 5A and Figure S2 in Supporting Information File 1), showing that CiCPS-PS retains the same activity as those of AsCPS and AsPS. Interestingly, CiCPS-PS exhibits the relatively low sequence identity to AnCPS-PS (34%) and NfCPS-PS (39%), both of which synthesize **1** [13]. This observation may indicate that evolutional origins of CiCPS-PS and these enzymes are different or not closely related. As mentioned above, TCs are considered to be evolved by fusion and loss of domains. Following this model, CiCPS-PS might be ancestral and its duplication and subsequent loss of domains likely generate separate type TCs, such as AsCPS and AsPS (Figure 5B, model a). Alternatively, translocation and clustering of two bifunctional TCs instead of gene duplication TCs can also be considered (Figure 5B, model b).

## Conclusion

Through genome mining of LRDs in fungi, we identified a unique pair of TCs in which domain organizations are distinct from those of known fungal TCs involved in the related pathways. Heterologous expression in *A. oryzae* and biochemical characterization of recombinant proteins showed that the identified enzymes are responsible for the biosynthesis of (−)-**1**. We also identified the bifunctional TC that could synthesize **1**. Since a set of enzymes retaining the same activity but having different domain organizations were discovered, these enzymes would be useful to discuss and further analyze the evolution of TCs in fungi.

## Supporting Information

File 1 Experimental part.

## Data Availability

All data that supports the findings of this study is available in the published article and/or the supporting information to this article.
