# Supplementary material for: Genome mining of labdane-related diterpenoids: Discovery of the two-enzyme pathway leading to (−)-sandaracopimaradiene in the fungus Arthrinium sacchari
Source: Beilstein J Org Chem. 2024 Apr 3;20:714–20. doi: 10.3762/bjoc.20.65 (PMC10999977; doi:10.3762/bjoc.20.65)
Supplement: File 1 — Experimental part. [file Beilstein_J_Org_Chem-20-714-s001.pdf]

BEILSTEIN JOURNAL OF ORGANIC CHEMISTRY

Supporting Information

for

Genome mining of labdane-related diterpenoids: Discovery of the two-enzyme pathway leading to (−)-sandaracopimaradiene in the fungus *Arthrinium sacchari*

Fumito Sato, Terutaka Sonohara, Shunta Fujiki, Akihiro Sugawara, Yohei Morishita, Taro Ozaki and Teigo Asai

Beilstein J. Org. Chem. **2024,** *20,* 714–720. doi:10.3762/bjoc.20.65

Experimental part

License and Terms: This is a supporting information file under the terms of the Creative Commons Attribution License (https://creativecommons.org/licenses/by/4.0). Please note that the reuse, redistribution and reproduction in particular requires that the author(s) and source are credited and that individual graphics may be subject to special legal provisions.
The license is subject to the *Beilstein Journal of Organic Chemistry* terms and conditions: (https://www.beilstein-journals.org/bjoc/terms)

Table of contents

SI-S2: General experimental procedure
SI-S2: Strains
SI-S2: Preparation of genomic DNA
SI-S2: Genome sequencing
SI-S2: Accession numbers
SI-S2: Construction of the *A. oryzae* transformant expressing *AsPS*, *AsCPS*, and *AsGGS*
SI-S3: Culture medium for the *A. oryzae* transformants
SI-S3: Cultivation of the transformants in CPS medium and HPLC analysis
SI-S3: Isolation of (−)-sandaracopimaradiene (**1**)
SI-S3: Protein expression and purification for in vitro assays
SI-S4: In vitro enzyme assays for AsPS and AsCPS
SI-S4: Construction of the *A. oryzae* transformant expressing *CiCPS-PS* and *AsGGS*
SI-S4: References
SI-S5: **Table S1**. Oligonucleotides used in this study.
SI-S6: **Table S2**. ^{1}H and ^{13}C NMR data of **1**.
SI-S7: **Figure S1.** Sequence alignment of fungal TCs involved in the biosynthesis of labdane-related diterpenoids.
SI-S8: **Figure S2.** MS spectra of the peaks shown in Figures 3, 4, and 5.
SI-S9: **Figure S3.** SDS-PAGE analysis of recombinant proteins with 10% polyacrylamide gel.
SI-S10: **Figure S4.** GC–MS analysis of the reaction by AsCPS and ObCPS_11g.
SI-S11: **Figure S5.** A cluster heatmap representation of cblaster analysis of the BGC containing *AsPS*, *AsCPS*, and *AsGGS*.
SI-S12: ^{1}H NMR spectrum of **1** (600 MHz, $CDCl_3$).
SI-S13: ^{13}C NMR spectrum of **1** (150 MHz, $CDCl_3$).

General experimental procedure

Analytical TLC was performed on silica gel 60 F254 (Merck) and RP-18 F254 (Merck). Column chromatography was carried out on silica gel 60 (70–230 and 40–50 mesh). The 600 MHz NMR spectra were recorded on a JEOL ECA-600 spectrometer (^{1}H NMR, 600 MHz; ^{13}C NMR, 150 MHz). Chemical shifts for ^{1}H and ^{13}C NMR are given in parts per million (δ) relative to tetramethylsilane (δ_H 0.00) and residual solvent signals (δ_C 77.0) for $CDCl_3$ as internal standards. HRMS–ES was measured on a JMS-700 MStation (JEOL Ltd.). GC–MS analyses were performed on a SCION 456-GC TQ system (Bruker) equipped with DB-5MS capillary column (0.25 mm× 30 m, 0.25 μm thickness; Agilent).

Strains

Escherichia coli DH5α was used for cloning and following standard recombinant DNA techniques. *E. coli* BL21(DE3) was used for the expression of histidine-tagged proteins. *Asperillus oryzae* NSAR1 (*niaD*$^-$, *sC*$^-$, Δ*argB*, *adeA*$^-$) and *A. oryzae* NSPlD1 (*niaD*$^-$, *sC*$^-$, *ΔpryG*, *ΔligD*) were used as the host for fungal expression. *Arthrinium sacchari* (*Apiospora sacchari*) Kumo-3 and *Ar. sacchari* MPU169, *Cordyceps indigotica* (*Metarhizium indigoticum*) NBRC 100684 was used for genomic DNA preparation and draft genome sequencing.

Preparation of genomic DNA

The genomic DNA of *Ar. sacchari* Kumo-3, *Ar. sacchari* MPU169, and *C. indigotica* NBRC 100684 were prepared as described previously.[S1]

Genome sequencing

Genome sequencing of *Ar. sacchari* Kumo-3 was conducted by Hokkaido System Science Co., Ltd. using Hiseq system (Illumina) and the obtained reads were assembled using Velvet 1.2.10.[S2] Genome sequencing of *Ar. sacchari* MPU169, and *C. indigotica* NBRC100684 was conducted by Macrogen Japan Corp. using Novaseq6000 system (Illumina). Assembly of obtained reads was conducted by Research Institute of Bio-System Informatics (Tohoku Chemical Co., Ltd.) using SPAdes 3.14.1.[S3]

Accession numbers

The sequences of identified genes have been deposited in the DNA Data Bank of Japan (DDBJ) with the following accession numbers;
LC795829: AsGGS from *Ar. sacchari* Kumo-3
LC795830: AsGGS from *Ar. sacchari* MPU169
LC795831: AsPS from *Ar. sacchari* Kumo-3
LC795832: AsPS from *Ar. sacchari* MPU169
LC795833: AsCPS from *Ar. sacchari* Kumo-3
LC795834: AsCPS from *Ar. sacchari* MPU169
LC795835: CiCPS-PS
LC795836: CiGGS

Construction of the *A. oryzae* transformant expressing *AsPS, AsCPS,* and *AsGGS*

The genes were amplified using genomic DNA of *Ar. sacchari* Kumo-3 by PrimeSTAR® MAX DNA Polymerase (TAKARA) with primers collected in Table S1. *Escherichia coli* DH5α were used for cloning, following standard recombinant DNA techniques. Fungal expression plasmid pUARA2[S4] possessing the α-amylase promoter (*amyB*) of *A. oryzae* and auxotrophic marker *argB* of *A. nidulans* respectively was used. The *AsPS* gene was amplified with the primers AS-TC1-IFpUKpnI-FW and AS-TC1-IFpUKpnI-RV. The *AsCPS* gene was amplified with the primers AS-TC2-IFpUKpnI-FW and AS-TC2-IFpUKpnI-RV. The *AsGGS* gene was amplified with the primers AS-GGPP-IFpUKpnI-FW and AS-GGPP-IFpUKpnI-RV. The PCR products were purified. The *AsPS, AsCPS,* and *AsGGS* were cloned into pUARA2 which had been digested with KpnI and NotI to yield pUARA2-*AS-TC1-TC2-GGPP*. The constructed plasmid was used for transformation of *A. oryzae* NSAR1 according to the procedures described previously.[S1]

Culture medium for the *A. oryzae* transformants
CPS medium: 1.75% Czapek–Dox Broth, 0.17% meat peptone, 0.17% soy peptone, 0.17% casein peptone, 1.0% soluble starch, 0.5% maltose monohydrate in 60/150 mL distilled water.

Cultivation of the transformants in CPS medium and HPLC analysis
The transformants were cultivated on a selection agar plant at 30 °C and its mycelia were inoculated in CPS medium and incubated at 30 °C for 5 days. The freeze-dried mycelium (30 mg) was extracted with EtOAc (950 µL) and MeOH (50 µL). After being centrifuged at 13,500 rpm for 15 min, the extract was transferred to a new tube and washed by H_2O. The organic layer was concentrated in vacuo. The residue was then dissolved in *n*-hexane and injected into GC–MS.

GC–MS conditions: The injector temperature was 250 °C. Each sample was injected to the column at 70 °C in the splitless mode. After 5 min isothermal hold at 70 °C, the column temperature was increased by 20 °C min^{-1} to 280 °C, then kept at this temperature for 10 min. The flow rate of the helium carrier gas was set at 1.20 mL min^{-1}.

Isolation of (−)-sandaracopimaradiene (1)
The transformant harboring *AsGGS*, *AsPS*, *AsCPS*, and the one P450 gene in the franking region was cultivated in CPS medium (2.4 L; 150 mL × 16) at 30 °C for 5 days. After the fermentation, the culture media and the mycelia were separated by filtration. The mycelia were lyophilized and extracted by EtOAc containing 10% of MeOH two times to give crude extract (1.3 g). The mycelial extract was dissolved in EtOAc (80 mL), washed with an equal volume of H_2O, and concentrated in vacuo. The extracts were subjected to silica gel column chromatography to give **1** (10.5 mg).

(–)-Sandaracopimaradiene (**1**)
$[\alpha]_D^{24}$ –14.3 (c 0.97, $CHCl_3$); HRMS (EI) *m*/*z*: $[M]^{+\cdot}$ Calcd for $C_{20}H_{32}$ 272.2499; Found 272.2494; ^1H and ^{13}C NMR data are summarized in Table S2.

Protein expression and purification for in vitro assays
AsPS and AsCPS were expressed in *E. coli* as recombinant proteins fused with N-terminal hexa-histidine tag. To construct plasmids for in vitro assay, protein coding sequences were amplified from the genomic DNA of *Ar. sacchari* Kumo-3 by PCR with the primer sets, AS-TC1_pET28a_NdeI-FW and AS-TC1_pET28a_EcoRI-RV for AsPS, and AS-TC2_pET28a_NdeI-FW and AS-TC1_pET28a_EcoRI-RV for AsCPS, respectively. The amplified DNA fragments were cloned into pET28a(+) using In-Fusion® Snap Assembly Master Mix (Takara Bio Inc.) to yield pET28a(+)-AsPS and pET28a(+)-AsCPS.
The *E. coli* BL21(DE3) cells harboring the plasmid pET28a(+)-AsPS or pET28a(+)-AsCPS were cultured in TB medium containing 30 µg/mL kanamycin at 37 °C to an OD600 of 0.4–0.6. After cooling the culture to 0 °C, isopropyl-1-thio-β-D-galactopyranoside (IPTG) was added at the final concentration of 0.1 mM to induce protein expression. The cultivation was further continued at 18 °C for 20 h. All the following procedures were performed at 4 °C. For purification, the cells were collected by centrifugation at 6,000*g* and resuspended in the lysis buffer (20 mM Tris-HCl (pH 7.6), 500 mM NaCl, 20 mM imidazole (pH 7.6)). The cells were disrupted by sonication, and the lysate was centrifuged at 15,000*g* for 15 min. The supernatant was loaded onto a Ni-Sepharose 6 Fast Flow (Cytiva) column equilibrated with the lysis buffer. After washing the resin with the same buffer, proteins were eluted with the elution buffer (20 mM Tris-HCl (pH 7.6), 500 mM NaCl, 250 mM imidazole (pH 7.6)). The buffer was replaced to 50 mM Tris-HCl (pH 7.6) containing 300 mM NaCl by PD-10 Desalting Column (PD-10).

In vitro enzyme assays for AsPS and AsCPS

Typical conditions for enzymatic reactions were as follows: a reaction mixture (300 μL of 50 mM Tris buffer, pH 7.6) containing 2 mM $MgCl_2$, 100 μM GGPP, 5 μM AsPS, 5 μM AsCPS was incubated at 30 °C overnight. The reaction mixture was extracted with 200 μL of hexane three times and concentrated in vacuo. The extract was dissolved in 100 μL of hexane and analyzed by GC–MS.

For hydrolysis of pyrophosphate group, the reaction mixture was extracted with *n*-BuOH and concentrated in vacuo. The extract was dissolved in the buffer containing 60% MeOH, 10 mM sodium acetate (pH 5.0), acid phosphatase (0.13 units, Sigma Aldrich). After the incubation at 37 °C overnight, the reaction mixture was extracted with hexane as described above.

For the reaction with ObCPS_11g, the cell-free extract of *E. coli* BL21(DE3) harboring *ObCPS_11g* was added to the reaction mixture. In the typical conditions, 220 μL of the extract dissolved in 50 mM Tris-HCl (pH 7.6) containing 300 mM NaCl was added to the 300 μL-scale reaction mixture.

Construction of the *A. oryzae* transformant expressing *CiCPS-PS* and *AsGGS*

The genes were amplified using genomic DNA of *C. indigotica* NBRC 100684 and *Ar. sacchari* MPU169 by KOD One® PCR master mix (TOYOBO CO., Ltd.) with primers collected in Table S2. *E. coli* DH5α were used for cloning, following standard recombinant DNA techniques. The ***CiCPS-PS*** gene was amplified with the primers CTC_fwd and CTC_rev. The linker containing terminator of *amyA* gene and promoter of *enoA* gene was amplified from pDP601C with the primers TamyA-PenoA-Insert-Fw and TamyA-PenoA-Insert-Rv. The amplified fragments and pDP601 digested with SmaI were assembled by NEBuilder HiFi DNA assembly master mix (New England Biolab) to yield pDP601:: ***CiCPS-PS*** /*AsGGS*. The constructed plasmid was used for transformation of *A. oryzae* NSPlD1 by a hot spot knock-in method according to the procedures described previously.[S5]

References

(S1) Tsukada, K.; Shinki, S.; Kaneko, A.; Murakami, K.; Irie, K.; Murai, M.; Miyoshi, H.; Dan, S.; Kawaji, K.; Hayashi, H.; Kodama, E. N.; Hori, A.; Salim, E.; Kuraishi, T.; Hirata, N.; Kanda, Y.; Asai, T. Synthetic Biology-Based Construction of Biological Activity-Related Library of Fungal Decalin-Containing Diterpenoid Pyrones. *Nat. Commun.* **2020**, *11*, Article number: 1830.

(S2) Zerbino, D. R.; Birney, E. Velvet: Algorithms for *de Novo* Short Read Assembly Using de Bruijn Graphs. *Genome Res.* **2008**, *18*, 821–829.

(S3) Bankevich, A.; Nurk, S.; Antipov, D.; Gurevich, A. A.; Dvorkin, M.; Kulikov, A. S.; Lesin, V. M.; Nikolenko, S. I.; Pham, S.; Prjibelski, A. D.; Pyshkin, A. V.; Sirotkin, A. V.; Vyahhi, N.; Tesler, G.; Alekseyev, M. A.; Pevzner. P. A. SPAdes: A New Genome Assembly Algorithm and Its Applications to Single-Cell Sequencing. *J. Comput. Biol.* **2012**, *19*, 455–477.

(S4) Tagami, K.: Minami, A.; Fujii, R.; Liu, C.; Tanaka, M.; Gomi, K.; Dairi, T.; Oikawa, H. Rapid Reconstitution of Biosynthetic Machinery for Fungal Metabolites in *Aspergillus oryzae*: Total Biosynthesis of Aflatrem. *ChemBioChem* **2014**, *15*, 2076–2080.

(S5) Liu, C.; Minami, A.; Ozaki, T.; Wu, J.; Kawagishi, H.; Maruyama, J. I.; Oikawa, H. Efficient Reconstitution of Basidiomycota Diterpene Erinacine Gene Cluster in Ascomycota Host *Aspergillus oryzae* Based on Genomic DNA Sequences. *J. Am. Chem. Soc.* **2019**, *141*, 15519–15523.

(S6) Tungcharoen, P.; Wattanapiromsakul, C.; Tansakul, P.; Nakamura, S.; Matsuda, H.; Tewtrakul, S. Anti-Inflammatory Effect of Isopimarane Diterpenoids from *Kaempferia galanga*. *Phytother. Res.* **2020**, *34*, 612–623.

(S7) Tamura, K.; Stecher, G.; Kumar, S. MEGA11: Molecular Evolutionary Genetics Analysis Version 11 *Mol. Biol. Evol.* **2021**, *38,* 3022–3027.

(S8) Robert, X.; Gouet, P. Deciphering Key Features in Protein Structures with the New ENDscript Server. *Nucl. Acids Res.* **2014**, *42,* W320–W324.

(S9) Gilchrist, C. L.; Booth, T. J.; van Wersch, B.; van Grieken, L.; Medema, M. H.; Chooi, Y. H. Cblaster: A Remote Search Tool for Rapid Identification and Visualization of Homologous Gene Clusters. *Bioinform. Adv.* **2021**, *1*, vbab016.

Table S1: Oligonucleotides used in this study.

Primer name	DNA sequence (5' to 3')
AS-TC1-IFpUKpnI-FW	CCGGAATTCGAGCTCGATGATGACGGCAAAATACGC
AS-TC1-IFpUKpnI-RV	ACTACAGATCCCCGGCGATTTCCATTCCACGTACG
AS-TC2-IFpUKpnI-FW	CCGGAATTCGAGCTCGACAATGGATCTAACCAACGA
AS-TC2-IFpUKpnI-RV	ACTACAGATCCCCGGATGCGTCCAGTTGCTTGGTC
AS-GGPP-IFpUNotI-FW	TTTGAGCTAGCGGCCAACATGGCATTGGTTCAGGAC
AS-GGPP-IFpUNotI-RV	GTCACTAGTGCGGCCCATGTCGACATAGCTTCTGC
AS-TC1_pET28a_NdeI-FW	CGCGCGGCAGCCATATGACGGCAAAATACGC
AS-TC1_pET28a_EcoRI-RV1	GACGGAGCTCGAATTACCAGGAACTATGCCTTTGG
AS-TC2_pET28a_NdeI-FW	CGCGCGGCAGCCATATGGATCTAACCAACGATGCGAG
AS-TC2_pET28a_EcoRI-RV	GACGGAGCTCGAATTTTTGGTGCCTTAGCGTGTTCC
GGPPS1_fwd	GCTCCGGAATTCGAGCTCGGTACCCATGGCATTGGTTCAGGAC
GGPPS_rev	TATGTTTCATCATCGGGTACGACCCTCAGTCGTTGTCAATCTTG
TamyA-PenoA-Insert-Fw	GGGTCGTACCCGATGATG
TamyA-PenoA-Insert_Rv	GGGTTTGACGAGCTGC
CTC_fwd	ACCAATTCCGCAGCTCGTCAAACCCATGGGTCAACACAAACAC
CTC_rev	CCTTCACGAGCTACTACAGATCCCCTCAAGCTACTGGCTCCAC

Table S2: ^{1}H and ^{13}C NMR data of **1**.

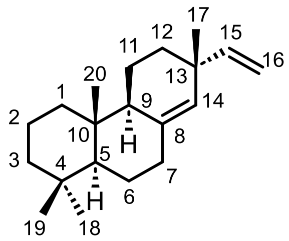

Position	^{1}H (mult, *J* in Hz) (ppm)[a]	^{1}H (mult, *J* in Hz) (ppm)[S6]	^{13}C (ppm)[a]	^{13}C (ppm)[S6]
1	1.01 (1H, m)	0.97 (1H, ddd, 8.0, 3.0, -)	39.4	39.4
	1.71 (1H, m)	1.71 (1H, m)		
2	1.72 (1H, m)	1.72 (1H, m)	18.8	18.8
	1.62 (1H, m)	1.62 (1H, m)		
3	1.18 (1H, br td, 13.2, 3.6, -)	1.18 (1H, br td, 13.1, 3.7, -)	42.2	42.2
	1.43 (1H, m)	1.43 (1H, m)		
4	-	-	33.3	33.3
5	1.03 (1H, m)	1.02 (1H, d, 2.0)	54.8	54.8
6	1.58 (1H, m)	1.58 (1H, m)	22.6	22.6
	1.29 (1H, dddd, 25.8, 13.2, 4.2, -)	1.28 (1H, dddd, 25.4, 13.0, 4.8, -)		
7	2.25 (1H, ddd, 14.4, 4.8, 1.8)	2.25 (1H, ddd, 14.0, 4.0, 2.0)	36.0	36.0
	2.04 (1H, tdt, 13.8, 6.0, 1.8)	2.05 (1H, tdt, 14.0, 6.0, -)		
8	-	-	137.3	137.3
9	1.69 (1H, m)	1.69 (1H, m)	50.7	50.6
10	-	-	38.3	38.3
11	1.42 (1H, m)	1.42 (1H, m)	19.1	19.0
	1.52 (1H, m)	1.52 (1H, m)		
12	1.48 (1H, m)	1.48 (1H, m)	34.6	34.6
	1.35 (1H, tdd, 12.6, 3.6, -)	1.35 (1H, tdd, 12.4, 4.1, -)		
13	-	-	37.4	37.4
14	5.21 (1H, s)	5.21 (1H, s)	128.5	128.5
15	5.78 (1H, dd, 17.4, 10.8)	5.77 (1H, dd, 17.0, 11.0)	149.2	149.1
16	4.88 (1H, dd, 10.8, 1.8)	4.88 (1H, dd, 11.0, 1.0)	109.9	110.0
	4.91 (1H, dd, 17.4, 1.8)	4.90 (1H, dd, 17.0, 1.0)		
17	1.04 (3H, s)	1.04 (3H, s)	26.0	26.0
18	0.88 (3H, s)	0.87 (3H, s)	33.8	33.8
19	0.85 (3H, s)	0.85 (3H, s)	22.1	22.1
20	0.80 (3H, s)	0.79 (3H, s)	15.0	15.0

[a]Experimental data in this study.

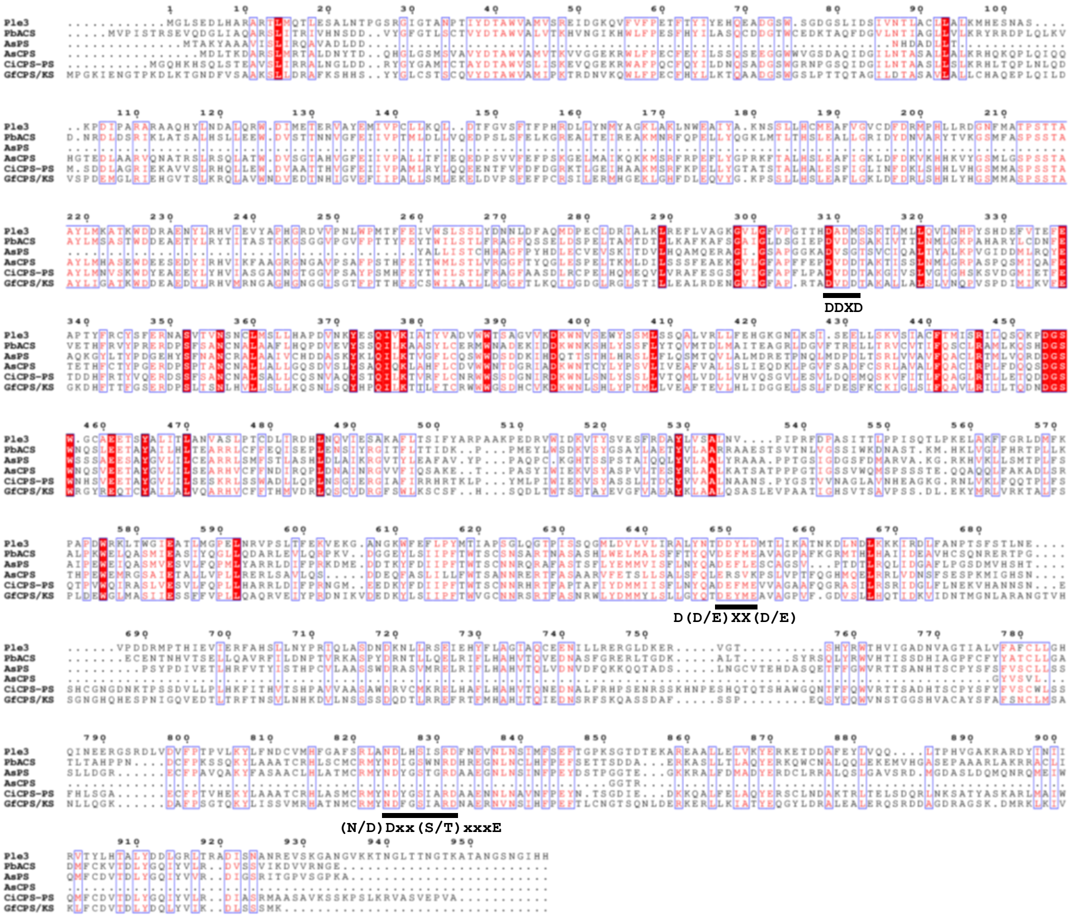

Figure S1: Sequence alignment of fungal TCs involved in the biosynthesis of labdane-related diterpenoids. The positions of three active center motifs are highlighted by bars. Three bifunctional TCs, Ple3 (Accession No. A0A6S6QR11) in pleuromutilin biosynthesis, PbACS (Accession No. Q96WT2) in the aphidicolin biosynthesis, and GfCPS/KS (Accession No. S0EA85) in gibberellin biosynthesis, are compared with the AsPS, AsCPS, and CiCPS-PS. After the sequences were aligned by MUSCLE using MEGA,[S7] the resulting alignment was depicted using ESPript 3.0.[S8]

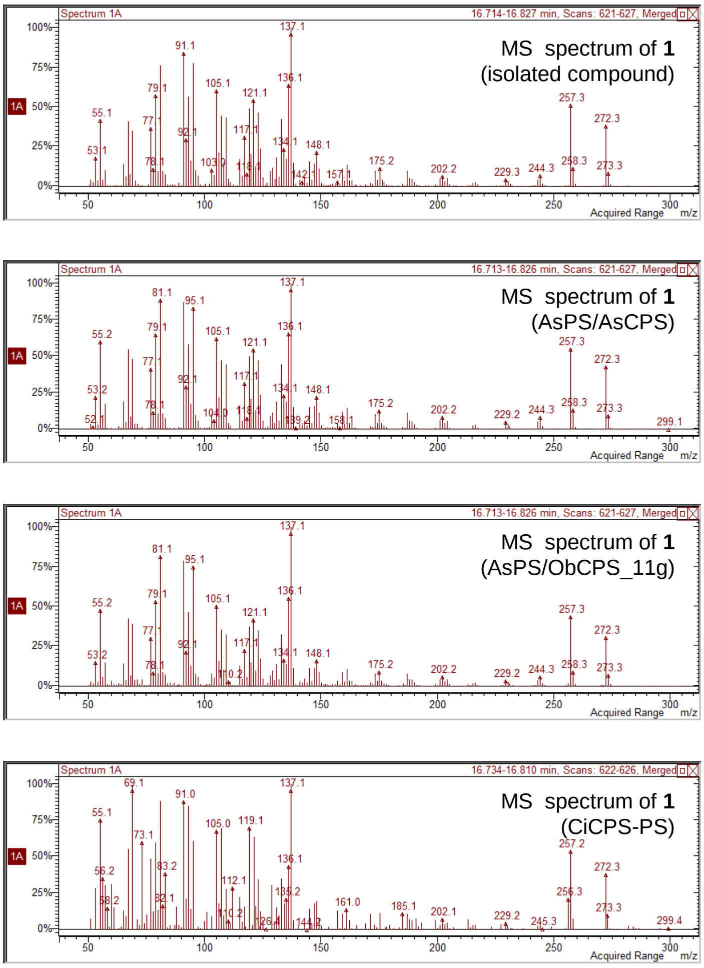

Figure S2: MS spectra of **1** shown in Figures 3, 4, and 5.

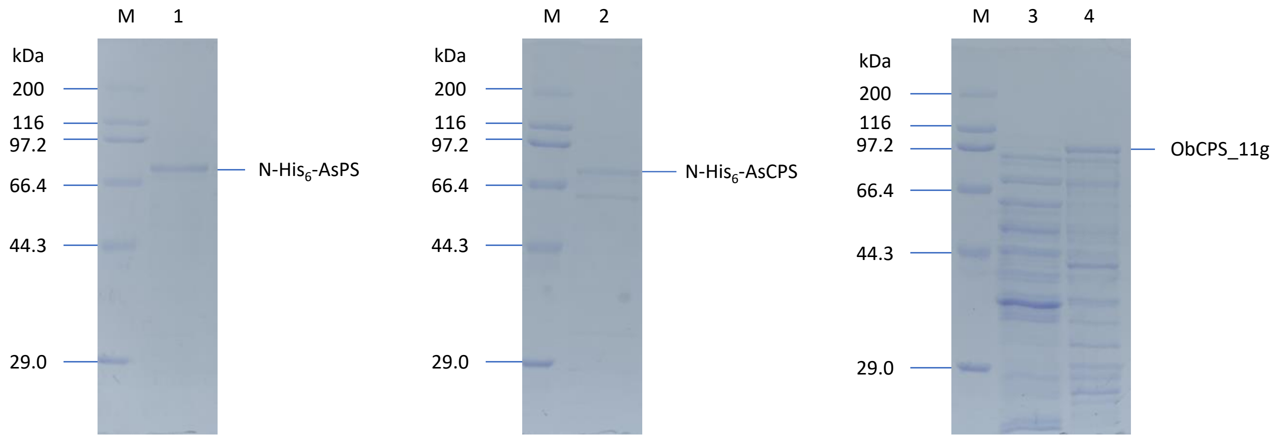

Figure S3: SDS-PAGE analysis of recombinant proteins with 10% polyacrylamide gel.
Lane 1: Purified N-His$_6$-AsPS. Lane 2: Purified N-His$_6$-CPS. Lane 3 and 4: Soluble proteins extracted from *E. coli* BL21(DE3) harboring ObCPS_11g gene (lane 3: 0 mM IPTG; lane 4: 0.5 mM IPTG). M: Protein Molecular Weight Marker (Broad) (Takara, Cat# 3452).

A

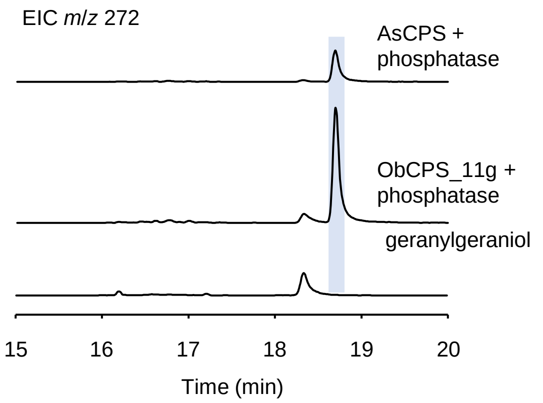

B

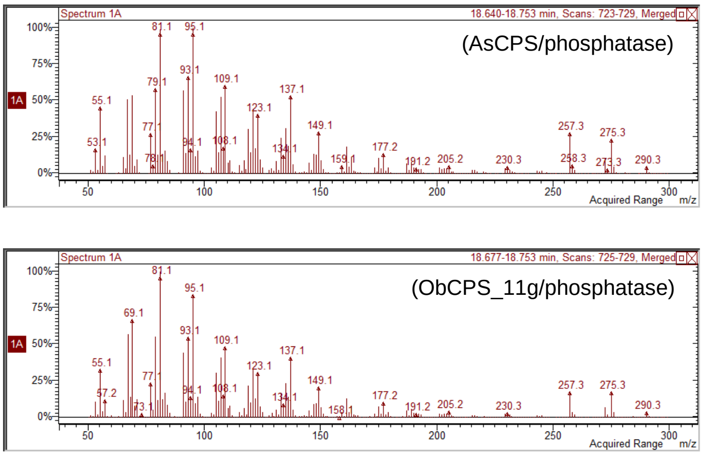

Figure S4: GC–MS analysis of the reaction by AsCPS and ObCPS_11g.
A) EICs at *m/z* 272 of the extracts from the reaction mixture of AsCPS and ObCPS_11g treated by acid phosphatase are shown. B) MS spectra of the peak at 18.7 min.

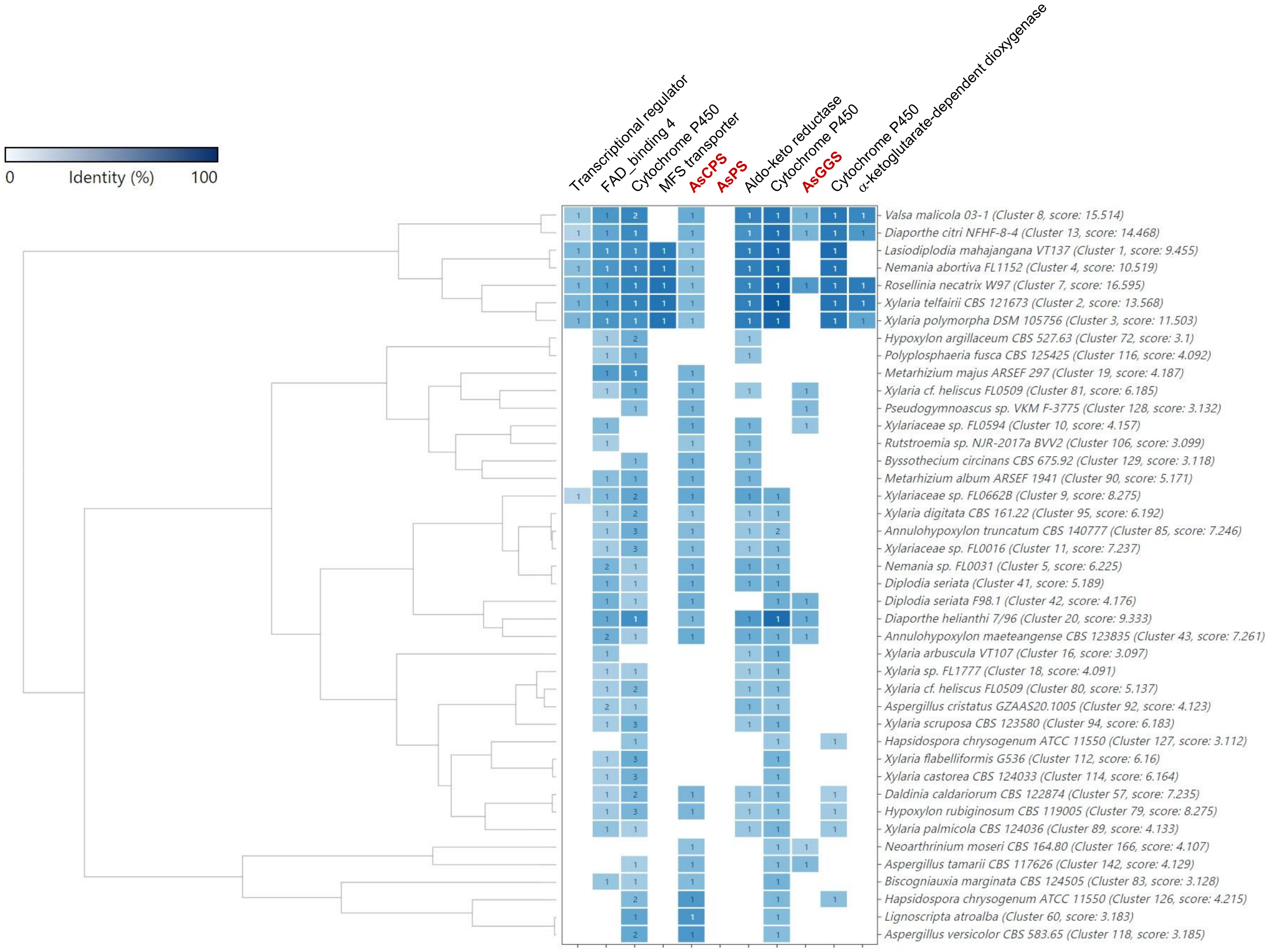

Figure S5: A cluster heatmap representation of cblaster[S9] analysis of the BGC containing *AsPS*, *AsCPS*, and *AsGGS*. The identified gene clusters were shown with names of fungal strains. Numbers in the blue boxes indicate the hit counts for each protein query. Dendrogram of hit clusters shown in the left side was generated from identity of hit clusters to query sequences. Note that only selected clusters are shown to reduce the number of BGC identified by the initial search.

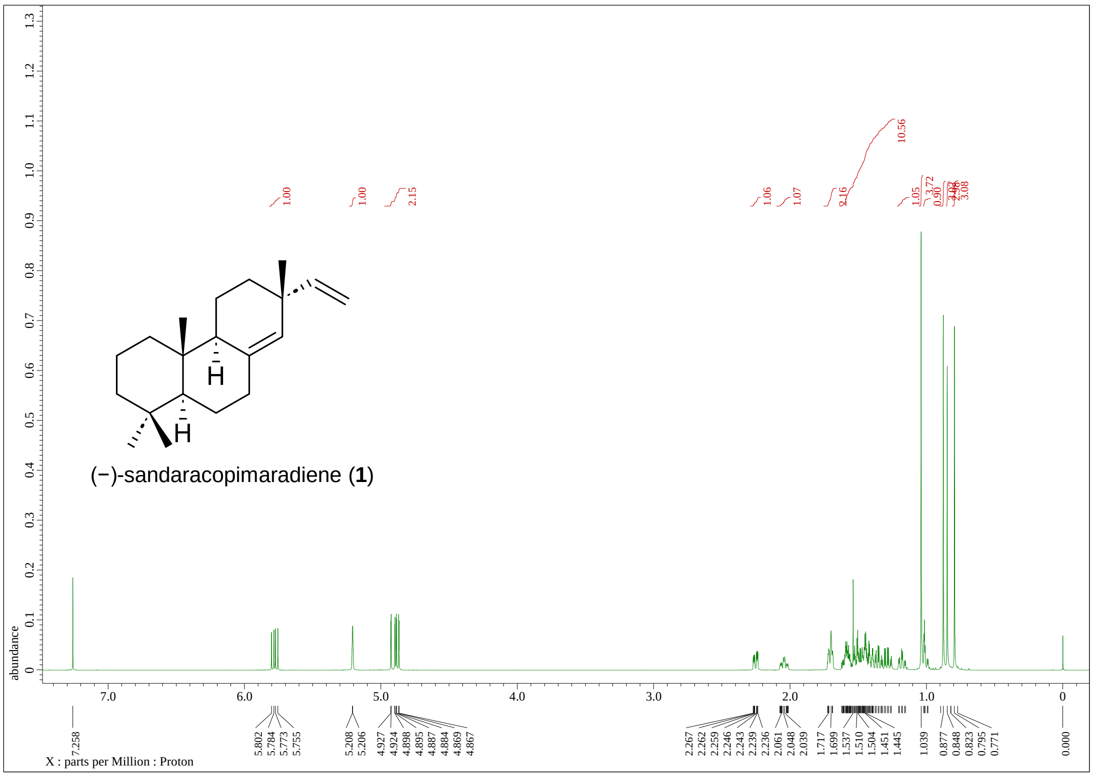

^{1}H NMR spectrum of **1** (600 MHz, $CDCl_3$).

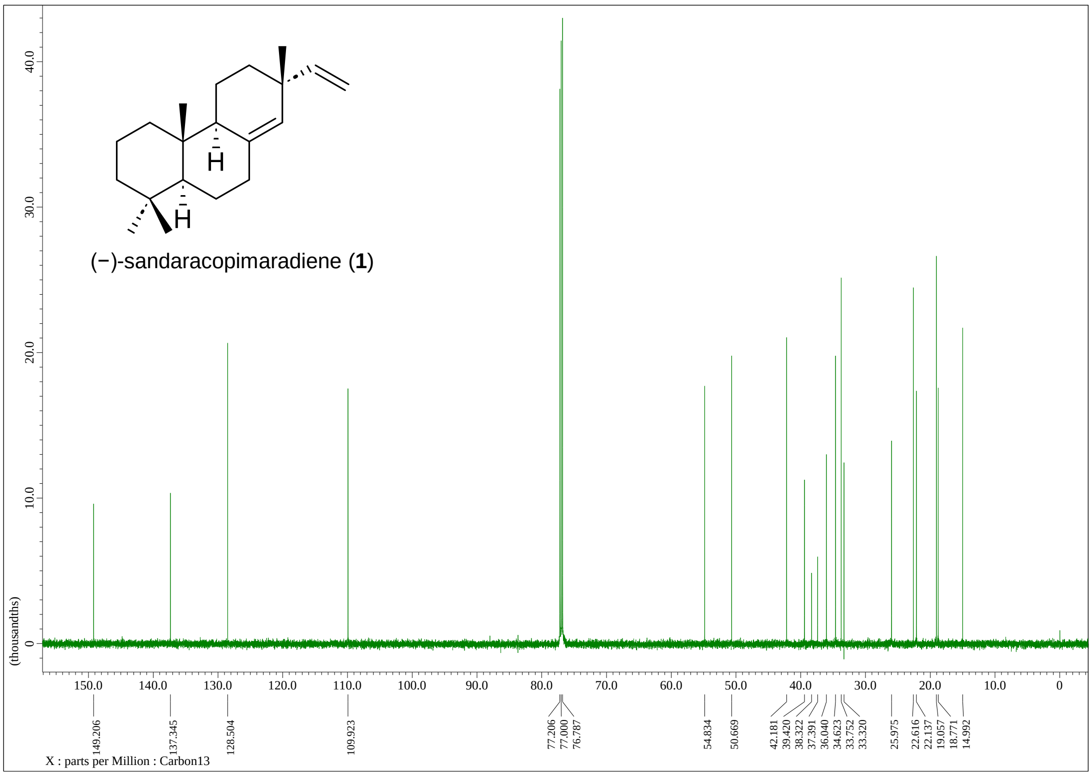

^{13}C NMR spectrum of **1** (150 MHz, $CDCl_3$).
